# Unveiling Humility in Emergency Medicine Chief Residents: A Thematic Exploration of Standard Letters of Evaluation

**DOI:** 10.5811/westjem.47058

**Published:** 2025-11-18

**Authors:** Abagayle Bierowski, Ridhima Ghei, Casey Morrone, Xiao Chi Zhang, Dimitrios Papanagnou

**Affiliations:** * Thomas Jefferson University, Department of Emergency Medicine, Philadelphia, Pennsylvania; † Sidney Kimmel Medical College, Philadelphia, Pennsylvania

## Abstract

**Introduction:**

Although humility is a key leadership trait linked to collaboration and trust, current residency application processes lack methods to identify it. By examining whether themes of humility appear in the Standardized Letters of Evaluation (SLOE) of medical students who later became emergency medicine (EM) chief residents, we sought to determine the presence of humility-related traits in SLOEs and explore their potential to inform the identification of applicants with leadership potential during residency selection.

**Methods:**

Two independent reviewers examined 104 SLOEs (52 chief, 52 non-chief) from 2015–2021, representing 43 students (21 who later assumed chief resident positions and 22 who did not) between 2018–2024 at a single academic EM residency program. A third reviewer resolved all coding disagreements. Reviewers deductively analyzed all written comments, targeting elements associated with humility as conceptualized by Tangney (2000) and Gruppen (2015). A SLOE was categorized as containing elements of humility if at least one clearly defined construct (such as openness to feedback, recognition of limitations, or concern for others) was identified. Sections of the data displaying the most convergence of humility elements underwent open coding, revealing emerging themes.

**Results:**

Nineteen of 21 (90.5%) chief residents had letters encompassing elements of humility compared to only 10 of 22 (45.5%) non-chief residents (P < .01). Openness was the most prominent element noted, followed by the need to make changes in performance, concern for others, and confidence. Further analysis of comments that highlighted humility uncovered several other themes including commitment and advocacy, eagerness to learn and improve, and maturity and responsibility.

**Conclusion:**

This study highlights specific humility-related traits noted in the Standard Letters of Evaluation of fourth-year medical students who later became chief residents in emergency medicine, offering preliminary insights into how qualitative evaluation tools may capture characteristics associated with future leadership roles.

## INTRODUCTION

Leadership in medicine requires more than clinical expertise; it demands qualities such as adaptability, collaboration, and emotional intelligence.^1^ Humility, often defined as an accurate self-assessment, recognition of one’s limitations, openness to new ideas, and appreciation of others’ contributions, is increasingly recognized as a cornerstone of effective leadership. Described as “the medical virtue most difficult to understand and practice,” humility is far from a sign of weakness. Instead, it reflects self-confidence, self-awareness, and the ability to transcend ego for the benefit of a team or system.^2^–^4^ According to leadership research, humility in leaders is characterized by their ability to maintain an objective view of themselves, value the strengths and contributions of others, and remain open to new ideas and feedback. Humble leaders demonstrate a willingness to self-reflect, acknowledge the capabilities of those they lead, and show a strong interest in learning from others’ perspectives and experiences. This openness and appreciation foster collaboration and adaptability, which are critical traits in effective leadership.^5^,^6^ In medicine, humility fosters a culture of empathy, collaboration, and continuous self-reflection, making it indispensable for both clinicians and leaders.

Although humility is valued in residency selection and leadership, it is not explicitly measured or surfaced in most formal evaluation tools, making it challenging to assess in residency applicants. Most residency selection processes focus on measurable achievements such as academic performance and clinical skills, with little emphasis on intangible traits such as humility. These behaviors are rarely included in formal evaluation rubrics, and in the absence of specific prompts, opportunities to capture humility through narrative comments may be overlooked. To our knowledge, there is no published literature directly evaluating how humility is assessed in residency applicants; however, anecdotally, it is widely regarded as a desirable yet difficult trait to identify during the application process, particularly in the absence of explicit prompts or structured tools. As a result, the absence of established methodologies for systematically identifying themes of humility in residency applicants presents an opportunity for improvement in graduate medical education, especially for programs seeking candidates who excel clinically and embody traits suited for future leadership roles.

While humility is not always explicitly considered in leadership selection, it is frequently cited as a core quality of effective leaders who are self-aware, open to feedback, and capable of fostering team-based success.^7^–^9^ In academic medicine, the appointment of chief residents is often one of the earliest formal recognitions of leadership potential. Although selection criteria vary across institutions, chief residents are typically chosen based on traits that frequently overlap with dimensions of humility: perceived maturity; reliability; emotional intelligence; and the ability to lead peers.^10^ We acknowledge that not all chief residents are necessarily more humble than their peers, and our study does not seek to establish that those selected possess greater humility.

Rather, in this study we operated on the assumption that the selection of chief residents reflects an endorsement of leadership potential that may have begun to emerge prior to residency and been further cultivated during training. We use the term “emerging” to refer to qualities that are not yet fully formed but are beginning to be expressed and recognized, such as openness to feedback, self-awareness, and maturity. While some of these attributes may be observable in Standardized Letters of Evaluation (SLOE) submitted during the emergency medicine (EM) residency application process, others may develop over time through mentorship, feedback, and clinical experience. This assumption provides a meaningful lens through which to explore whether humility-related behaviors are identifiable earlier in training.

**Population Health Research Capsule**

What do we already know about this issue?

*Leadership selection in residency is common, but the characteristics influencing chief resident selection are poorly understood.*

What was the research question?

*How is humility reflected in the Standard Letters of Evaluation (SLOE) of applicants who later become chief residents in emergency medicine?*

What was the major finding of the study?

*90.5% of chief residents vs 45.5% of non-chiefs had humility traits in their SLOEs (P < .01).*

How does this improve population health?

*Understanding valued leadership traits such as humility may help shape more intentional, equity-focused selection and development of residents.*

While it may be intuitive that applicants who display desirable traits in SLOEs could eventually hold leadership roles, this study adds a theory-driven, qualitative approach to identifying one such trait (humility) using established conceptual models. In doing so, it provides preliminary insight into how humility-related behaviors may be expressed in real-world evaluations and offers a foundation for future research on evaluating intangible leadership traits in residency candidates.

## METHODS

As emergency physicians with firsthand knowledge of residency training and the chief resident selection process, we acknowledge the importance of reflexivity in qualitative research. The research team includes three women and two men, all of whom are practicing physicians. One team member is currently pursuing a master’s degrees in education, two have a Master of Health Professions Educations degree, one holds a Master of Science degree, and one has earned an EdD. All authors had substantial training in qualitative methods, and several have served as chief residents in their own programs, offering perspective on the expectations and selection processes commonly associated with the chief resident role. Importantly, three of the five authors were not affiliated with the institution during the selection period of the chief residents included in this study, and the remaining two authors held positions in undergraduate medical education that do not involve or influence graduate medical education decisions (including chief resident selection). These factors helped reduce the risk of bias related to participant selection or interpretation of findings. Additionally, we had no conflicts of interest and received no funding to conduct this research.

We conducted a retrospective review of SLOEs of medical students from 2015–2021 who matched into EM and later were selected for chief resident positions from 2018–2024 at a single-site, tertiary academic EM residency program. This range was selected to include all chief residents from our EM program for whom complete, archived SLOEs were available at the time of study initiation. This study adhered to the standards for reporting qualitative research (SRQR), which are synthesized recommendations that aim to improve the transparency of all aspects of qualitative research (see Appendix).^11^ We chose the SRQR because it addresses critical aspects of qualitative research, including reflexivity, study design, data analysis, and reporting, making it well-suited for the methodology used in this project.

### Study Design

We adopted a retrospective, qualitative study design guided by a constructivist framework, which posits that learning is an active process where individuals construct knowledge through their experiences to prioritize an in-depth examination of historical SLOEs to uncover emerging themes of humility. To mitigate potential bias during qualitative analysis, our dataset of SLOE excerpts intentionally excluded demographic information, including sex, age, and graduation year (Table 1) during the analysis. We acknowledge that certain narrative elements, such as descriptions of unique experiences, may still carry identifiable details; however, our focus was on reducing the influence of known demographic characteristics on data interpretation.

### Study Setting and Population

Conducted within the context of a single-site, academic EM residency program, this study focused on a distinct population: medical students who, between 2015–2021, matriculated into our residency program and were elected to chief resident positions. Our program is a postgraduate year (PGY) 1–3 program within a Level 1, urban, academic medical center in the Northeast. Each class consists of 17 residents, with three chief residents selected each year from the PGY-3 class. Selections are made through a hybrid process consisting of application review, interview scores, professionalism review, and voting by residents and faculty members. Chief residents represent a useful population for exploring the potential early presence of humility-related behaviors, for they are widely regarded as individuals who demonstrate traits that align with key dimensions of humility such as self-awareness, openness to feedback, and respect for others.^10^

### Study Protocol

**Figure 1:**
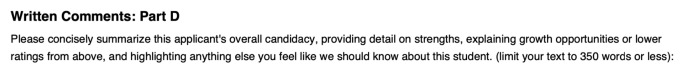
Part D of the Standardized Letter of Evaluation written comments section used to assess emergency medicine applicants: context for thematic analysis of humility in chief residents.

A total of 104 de-identified SLOEs underwent qualitative analysis. This included 52 SLOEs written for 21 medical students who later assumed chief resident roles, and 52 SLOEs from a randomly selected cohort of students who did not go on to become chief residents. Each resident had between one and four SLOEs included, yielding a total of 104 SLOEs (52 chief, 52 non-chief) in the final analysis. Our analysis was solely focused on the open-ended, qualitative “written comments” section that appears at the end of the SLOE document (Figure 1).

Since the qualitative section has been a consistent component of all versions of the SLOE, we were able to reliably extract qualitative data across multiple years. Two designated members of the research team independently reviewed and deductively coded all SLOEs, guided by the conceptual frameworks of Tangney (2000) and Gruppen (2015). A third team member adjudicated all coding discrepancies to maintain consistency across the dataset. This study was reviewed and exempted by the institutional review board at our university.

### Measurements and Key Outcome Measures

The primary focus of this study was to identify and characterize humility-related content within the narrative, open-ended portions of SLOEs. The key outcome measure was the presence of language or descriptions that aligned with established humility constructs, including self-awareness, openness to feedback, appreciation of others, and recognition of personal limitations. A SLOE was categorized as containing humility-related attributes if at least one of these predefined constructs was clearly identified in the narrative comments. Additional outcomes included the types and frequency of these humility-related elements, as well as any recurring patterns in how they were described.

Using a constructivist approach, we conducted a qualitative content analysis of SLOE narratives, emphasizing the interpretive and contextual nature of the data. The analysis began with a deductive coding framework based on established humility constructs, followed by open coding to identify emergent themes beyond the initial framework. Manual coding was performed independently by members of the research team without the use of qualitative analysis software. As this was an exploratory qualitative study, we did not perform statistical testing, and sample size was determined by the bounded study population rather than thematic saturation.

## RESULTS

Our investigation uncovered a significant prevalence of humility-related attributes in the chief residents’ SLOEs: 19 of 21 (90.5%) chief residents had letters encompassing elements of humility, compared to only 10 of 22 (45.5%) non-chief residents (*P* < .01, Fisher exact test). Humility-related attributes were significantly more common in SLOEs written for students who became chief residents. When evaluating individual SLOEs, humility appeared in 31 of 52 (59.6%) chief resident SLOEs vs 13 of 52 (25.0%) non-chief SLOEs (χ^2^ (1, N = 104) = 12.76, *P* < .001).

Openness was the most prominent element noted in chief resident SLOEs, followed by the need to make changes in performance, concern for others, and confidence (Table 2). These four elements represent how the predefined humility constructs—self-awareness, openness to feedback, appreciation of others, and recognition of personal limitations—manifested in the narrative data. For example, “openness” maps directly to openness to feedback, while “need for ongoing performance improvement” reflects both recognition of limitations and self-awareness. “Concern for others” aligns with appreciation of others. Although “confidence” was not part of our original coding framework, it frequently co-occurred with humility-related language and was, therefore, included as a contextual theme relevant to the overall construct.

Each chief resident in the study had between 1–4 SLOEs included in the dataset. A chief resident was categorized as having humility-related attributes if at least one SLOE contained a clearly identifiable construct consistent with our predefined coding framework. This inclusive threshold was chosen given the variability in SLOE count and length across individuals. Of the 21 chief residents, 19 (90.5%) met this threshold. The four primary elements were identified across these 19 individuals as follows: openness (N = 15); need for ongoing performance improvement (N = 13); concern for others (N = 9); and confidence (N = 9). These elements were not mutually exclusive and often co-occurred within the same SLOE or across multiple letters. Each element was counted once per chief resident, meaning that if a resident had more than one SLOE containing the same element, that element was only counted once in the total (N). Among the three chief residents whose SLOEs did not meet the inclusion threshold, no identifiable humility-related constructs were coded. While some comments reflected general positivity or professionalism, they did not include language that clearly aligned with the predefined elements of humility as operationalized in our coding framework.

### Openness

Openness was manifested in various ways across the letters, indicating a consistent pattern of behavior among the prospective chief residents. For instance, a recurring element in the letters highlighted the candidates’ ability to interact effectively with diverse patients and tailor discharge instructions to varying levels of understanding within the patient population. Evaluators frequently emphasized the candidates’ curiosity and dedication to learning, as one described: “Inquisitive and asks pertinent and appropriate questions in order to further his knowledge. [He/She] wants to learn as much as [he/she] possibly can.” Additionally, the language used by the evaluators consistently underscored the candidates’ willingness to collaborate, consider alternative approaches, and “ability to adapt,” reflecting an openness that transcended individual perspectives. Another dimension of openness is observed in candidates’ proactive and eager attitudes toward learning and improvement, demonstrated by their involvement in various procedures, volunteering during crises, and an overarching eagerness to participate in learning opportunities.

### Need for Ongoing Performance Improvement

The SLOEs consistently illuminate candidates’ awareness of the need for ongoing performance improvement, underscoring another core theme of humility. One key strength identified in future leaders is their constant search for ways to improve, coupled with a genuine care for the world around them. Letters highlighted the improvement trajectory of several students, demonstrating a proactive attitude toward professional development. For example, “stays late to learn more,” “improves with each shift, starting to see the big picture,” and “eager to continue improving.” These comments signify a maturation process and a commitment to evolving in their role. Similarly, SLOEs consistently expressed student eagerness and receptiveness to feedback, as well as the ability to anticipate needs, demonstrating keen observational traits and an adept understanding of the dynamic environment in EM. The humility to recognize limitations and seek clarification through questions is evident, emphasizing a commitment to continuous learning and improvement.

### Concern for Others

Another common thread in letters of chief residents was the expression of concern for others, often manifested as candidates going above and beyond to tailor care to the understanding of patients and advocating for their well-being. Instances of volunteering during crises, such as the COVID-19 pandemic, underscore a genuine commitment to patient care during challenging times. Compassion and empathy are consistently highlighted, with comments such as “truly cares about the world around [him/her]” and “great advocate for [his/her] patients.” Other comments spoke to students’ consistent re-evaluation of patients and independent discussions on care, stating, “[He/she] would consistently re-evaluate patients and independently discuss care with them.” Another wrote, “Strong empathetic personality which allows to interact on a more humane level with his patients.” Students’ consistent re-evaluation of patients, independent discussions on care, and taking true ownership of patient outcomes not only underscore their empathetic approach and dedication to individualized patient well-being but also reflect a humble commitment to continuous improvement and a genuine recognition of the complexities inherent in patient care.

### Confidence

Although not traditionally conceptualized as a core component of humility, confidence frequently co-occurred with humility-related behaviors in chief resident SLOEs. Letters often described a balanced confidence that was paired with openness to feedback, eagerness to learn, or self-awareness, supporting rather than contradicting the presence of humility. For this reason, confidence was included as a contextual element reflecting how humility was often expressed in conjunction with leadership readiness. In their SLOEs, chief residents’ confidence was noted through interactions with consultants, primary care physicians, and patients, where they are described as outspoken, persuasive, and committed to their patients’ well-being. Comments include “strikes an incredible balance of confidence with enthusiastic learner,” “confident but humble,” and “confident, outspoken, and naturally persuasive with consultants.”

### Other Themes

Comment analysis revealed several other minor themes of humility in chief resident letters, including the ability to ask for help/guidance; maintaining perspective; acknowledgment of shortcomings; commitment and advocacy; eagerness to learn and improve; initiative and resourcefulness; teachability and adaptability; empathy and compassion; maturity and responsibility; proactivity and ownership; enthusiasm and positivity; continuous improvement; and dedication to patient care. These themes were identified exclusively in the SLOEs of the 19 chief residents who met our threshold for humility-related content; none were present in the SLOEs of the three excluded individuals. Given the exploratory and qualitative nature of this phase of analysis, our intent was to describe the thematic landscape of humility-related language rather than to quantify the frequency of each individual code.

## DISCUSSION

Our study was grounded in two of the most well-established and frequently cited definitions of humility in the literature: those proposed by Tangney (2000) and Gruppen (2015).^12^,^13^ By incorporating both frameworks, we aimed to comprehensively operationalize the construct of humility and reduce the likelihood of missing key elements within the data. The humility-related themes identified within SLOEs offer a nuanced perspective on the qualities associated with effective and compassionate leadership in EM. While “hard” skills such as clinical knowledge and procedural competencies are vital, the prominence of “soft” skills such as commitment, enthusiasm, teachability, empathy, and proactivity underscores their pivotal role in shaping chief residents. These qualities collectively form a comprehensive framework for assessing humility, emphasizing its dynamic and interconnected nature.

Our findings that most chief residents had several elements of humility threaded through their SLOEs align with existing literature on humility in leadership and medicine. Owens and Hekman discuss emergent humility in the leadership model, emphasizing the importance of humility in leader-follower relationships and organizational development.^6^ Rego et al present an empirical study on the perceived impact of leaders’ humility on team effectiveness and encourage humility to be included in any authentic leadership agenda.^14^ Similarly, Collins and Stoller discuss the concept of “level 5 leadership,” which involves the co-occurrence of personal humility and an unwavering commitment to produce long-term results.^15^,^16^ Collectively, these models resonate with our study’s identification of openness, concern for others, and the need for ongoing performance improvement as prominent humility-related themes in the SLOEs of chief residents.

Recognizing humility as not only an important trait for leaders but also for successful clinicians, Wadhwa and Mahant illuminate the lived experiences of peer-nominated, excellent clinicians through a qualitative exploration of humility in medical practice. In their study, humility emerges as a key driver for excellence, playing a pivotal role in shaping positive relationships with patients and team members and fostering a collaborative, patient-centric healthcare environment.^3^ This underscores the consistent recognition of humility’s significance in medical leadership, aligning with our findings in chief residents’ SLOEs.

Moreover, humility in chief residents contributes to fostering effective teamwork and creating an inclusive atmosphere within the emergency department. Leaders who acknowledge their limitations set an example for continuous learning and improvement, inspiring a culture of humility among their peers and subordinates. This culture, in turn, can have a ripple effect that may enhance the overall dynamics of a medical team and ultimately lead to improved patient outcomes and a more resilient healthcare system.

Our findings highlight a noteworthy aspect of humility concerning its relationship with confidence, suggesting that these traits are not mutually exclusive but can coexist as complementary attributes. Specifically, our study suggests that confidence, when balanced with humility, may reflect a self-assured yet teachable mindset that can contribute to effective leadership. This interpretation aligns with Chiu et al, who explore how leader humility and team member characteristics influence shared leadership and team dynamics, and with Lombardero et al, who underscore the importance of cultural humility in medical education.^17^,^18^ While our investigation of chief residents’ SLOEs suggests a recurring balance of confidence and humility, further research is needed to better understand how these traits manifest in leadership roles. This study provides preliminary insights into the complex interplay between humility and confidence in EM leadership, offering a foundation for future exploration.

Additionally, while we identified humility-related traits in SLOEs written during medical students’ fourth year, it remains unclear whether these traits were further developed or reinforced during residency training. Existing literature suggests that having a growth mindset, closely aligned with humility, plays a critical role in creating adaptable leaders.^6^,^19^ This raises an important question about whether residency programs can actively nurture and enhance these traits, potentially shaping individuals into effective leaders over time. Future longitudinal studies could explore how traits like humility evolve and contribute to leadership development throughout residency.

It is also worth considering whether the humility-related elements described in the SLOEs represent authentic traits of the applicants or are instead emphasized by letter writers who recognize that qualities like teamwork and self-awareness are highly valued by residency programs. Narrative letters are subject to both intentional and unconscious bias, and the presence of these elements may reflect strategic framing by the author rather than objective traits of the applicant. Additionally, the observed humility-related elements may reflect qualities that were actively cultivated during residency rather than being present at the time of selection. As our study included only SLOEs from students who became chief residents, we cannot determine whether these traits were disproportionately present compared to peers who were not selected. Future research comparing SLOEs across different resident cohorts, and assessing traits longitudinally, would help clarify whether early humility indicators correlate with leadership trajectories.

Looking forward, the identification of humility-related themes in SLOEs holds promising implications for the practical aspects of residency program selection processes. Our study brings attention to the challenge faced by residency program directors in navigating through an extensive array of letters to discern applicant attributes, particularly for complex traits like humility. The conventional approach, reliant on explicit mentions of terms such as “humble,” may inadvertently overlook the subtler yet crucial aspects that contribute to effective and compassionate leadership. An opportunity to leverage the common threads of humility uncovered in our study certainly exists; establishing a systematic coding system based on these identified themes could provide a structured framework for residency program directors to assess and identify candidates with humility-related attributes. This approach could serve as a valuable tool, offering a more efficient and comprehensive means of evaluating a candidate’s potential for effective leadership.

As the healthcare landscape evolves with constant change and uncertainty, the importance of training future leaders who exhibit humility cannot be overlooked. Humble leaders are often more open to new ideas and perspectives, creating environments that support collaboration and problem-solving. In EM, leadership requires not only clinical expertise but also the ability to inspire confidence, foster teamwork, and adapt to challenges. While this study highlights the potential value of identifying humility-related traits during the residency selection process, further research is needed to better understand how these traits contribute to leadership effectiveness.

## LIMITATIONS

While our study provides valuable insights into the theme of humility within chief residents as identified in SLOEs, it is essential to acknowledge its inherent limitations. The SLOE, by nature, is a concise document, and the brevity of written comments poses a challenge in fully capturing the depth and nuances of “soft” skills, such as humility, that play a pivotal role in the selection of individuals for leadership roles. The constrained space may not allow for a comprehensive representation of the multifaceted aspects of humility, potentially limiting the richness of the data obtained from these evaluations.

Due to the conceptual nature of humility, some degree of thematic overlap and redundancy was expected in the results. While we identified humility-related themes and grounded our assumptions in existing leadership literature, it is important to note that not all chief residents may possess humility as a defining trait. This study was not designed to establish causation or predictive relationships. Instead, it sought to explore whether humility-related traits could be observed in SLOEs and whether these narrative evaluations might offer meaningful qualitative insights.

We recognize the potential for bias in SLOEs and chief resident selection, as existing literature highlights disparities in how evaluations are written and interpreted, potentially influenced by implicit bias. To mitigate these concerns, we intentionally anonymized demographic information from our dataset and emphasized reflexivity in our qualitative analysis. We also acknowledge that many of the humility-related phrases identified in our analysis may appear frequently across SLOEs in general, not only in those belonging to eventual chief residents. This raises important questions about the specificity of these elements and their value in identifying future leaders. While our findings suggest that humility-related traits can be captured in narrative comments, further research is needed to determine whether these elements are more prevalent or described differently in evaluations of future leaders compared to their peers.

Our single-site study further limits generalizability. Chief resident selection criteria may vary across institutions and regions due to differences in organizational culture and institutional priorities. While our findings are specific to EM, the use of SLOEs as a standardized evaluation tool is unique to this specialty. Other fields, which rely on traditional letters of recommendation, may not capture similar qualitative data on humility. Future research should explore whether humility and other leadership traits can be systematically identified in evaluations across different specialties and contexts.

Finally, we acknowledge that humility is a dynamic trait that may evolve over time, particularly through leadership experiences such as serving as a chief resident. Our study captures a snapshot of perceived humility-related behaviors during the residency application process but does not evaluate whether these traits were sustained, enhanced, or further developed during training, nor does it follow these individuals longitudinally to assess how these traits evolve or persist through residency and beyond. Future longitudinal studies are needed to better understand how leadership roles may shape or reinforce characteristics such as humility over time.

These limitations highlight the need for cautious interpretation of our findings and underscore the complexity of evaluating “soft” skills such as humility. Future multisite studies incorporating diverse institutional contexts and longitudinal assessments will be crucial to further our understanding of how traits like humility contribute to leadership in medical training.

## CONCLUSION

Our findings emphasize the potential for residency educators, particularly those involved in application review and trainee mentorship, to recognize and foster qualities such as humility in students and trainees. However, further research is needed to better understand how these traits influence leadership selection and performance. While our study found openness to be the most frequently occurring humility-related element, it is difficult to determine how to interpret the presence of any single behavior, particularly within the context of a small, qualitative study. Meaningful insight is more likely to come from identifying a composite of multiple humility-related traits that are observed consistently across different evaluations and clinical settings. As the medical community continues to prioritize the development of well-rounded and empathetic leaders, these insights may help inform future efforts to refine the evaluation of residency candidates and explore how humility contributes to leadership in emergency medicine.

## Supplementary Information



## Figures and Tables

**Table 1:** Demographic and characteristics of Standard Letters of Evaluation.

	Chiefs (All)	Non-Chiefs (Random Sample)
Men	12	14
Women	9	8
Mean Age	27.71	27.86
Independent SLOEs	14	7
Group SLOEs	38	45
Mean Number of Rotations	2.48	2.36

*SLOE*, Standardized Letter of Evaluation.

**Table 2:** Key elements of humility identified in Standardized Letters of Evaluation for emergency medicine chief residents, with illustrative quotes.

Humility element	Illustrative quotes
Openness (N = 21)	“Inquisitive and asks pertinent and appropriate questions…to further [his/her] knowledge. [He/she] wants to learn as much as [he/she] possibly can.”
	“Proactive and eager to learn.”
	“[He/she] was extremely proactive and was always asking to see patients. [He/she] sought out learning opportunities and showed enthusiasm.”
	“Ability to adapt.”

Need for Ongoing Performance Improvement (N = 14)	“Eager to continue learning and improving. Engaged, eager to learn, and receptive to feedback and coaching.”
	“Strong desire to learn and improve throughout [his/her] shifts.”
	“Receptive to feedback and coaching.”
	“Improves with each shift, starting to see the big picture. Grew substantially.”

Concern for Others (N = 12)	“[He/she] truly cares about the world around [him/her].”
	“Showed [his/her] commitment to patient care during the COVID-19 crisis in March by volunteering.”
	“Compassion for patients.”
	“Strong empathetic personality which allows to interact on a more humane level with [his/her] patients.”

Confidence (N = 9)	“Strikes an incredible balance of confidence with enthusiastic learner.”
	“Confident, outspoken, and naturally persuasive with consultants, PMDs, and patients.”
	“Engaging with a positive and cheerful attitude.”
	“Energetic, eager, and engaged.”

*PMD*, primary medical doctor.
